# Fatigue Is Associated with Poor Sleep in People with Multiple Sclerosis and Cognitive Impairment

**DOI:** 10.1155/2014/872732

**Published:** 2014-03-05

**Authors:** Michelle H. Cameron, Vanessa Peterson, Eilis A. Boudreau, Ashley Downs, Jesus Lovera, Edward Kim, Garnett P. McMillan, Aaron P. Turner, Jodie K. Haselkorn, Dennis Bourdette

**Affiliations:** ^1^Department of Neurology, School of Medicine, Oregon Health & Science University, 3181 SW Sam Jackson Park Road L226, Portland, OR 97239, USA; ^2^Multiple Sclerosis Center of Excellence-West, Portland Department of Veterans Affairs Medical Center, Portland, OR 97239, USA; ^3^Portland Lung Clinic, Adventist Medical Center, Portland, OR 97216, USA; ^4^Department of Medical Informatics and Clinical Epidemiology, School of Medicine, Oregon Health & Science University, Portland, OR 97239, USA; ^5^Epilepsy Center of Excellence, Portland Veterans Affairs Medical Center, Portland, OR 97239, USA; ^6^Department of Neurology, School of Medicine, Louisiana State University Health Sciences Center-New Orleans, New Orleans, LA 70112, USA; ^7^National Center for Rehabilitative Auditory Research, Portland Veterans Affairs Medical Center, Portland, OR 97239, USA; ^8^Department of Rehabilitation Medicine, University of Washington, VA Puget Sound Health Care System, VA MS Center of Excellence-West, Seattle, WA 98108, USA; ^9^Department of Epidemiology, University of Washington, VA Puget Sound Health Care System, VA MS Center of Excellence-West, Seattle, WA 98108, USA

## Abstract

*Background*. Fatigue is the most common symptom in people with multiple sclerosis (MS). Poor sleep also occurs in this population. *Objective*. The objective of this study was to determine the relationship between fatigue and sleep quality in people with MS and cognitive impairment. *Method*. This cross-sectional study assessed relationships among fatigue, assessed with the Modified Fatigue Impact Scale (MFIS) and the Fatigue Severity Scale (FSS), sleep quality assessed with the Pittsburg Sleep Quality Index (PSQI), and demographics in 121 people with MS and cognitive impairment. *Results*. Fatigue was significantly correlated with poor sleep quality (MFIS: *F* = 15.60, *P* < 0.01; FSS: *F* = 12.09, *P* < 0.01). FSS scores were also significantly correlated with the PSQI subscore for daytime dysfunction and MFIS scores were significantly correlated with disability, age, and the PSQI subscores for sleep quality, sleep duration, and daytime dysfunction. *Conclusions*. This study demonstrates a relationship between fatigue and sleep quality in individuals with MS and cognitive impairment.

## 1. Introduction

MS-related fatigue, “the subjective lack of physical and/or mental energy that is perceived by the individual or caregiver to interfere with usual and desired activities” [1], is one of the most common symptoms in people with multiple sclerosis (MS), affecting up to 92% of individuals with the disease [2, 3]. Poor sleep and cognitive impairment are also common in MS [4]. Although fatigue and poor sleep have been found to be related in the general population of people with MS [5], the degree to which fatigue and sleep complaints overlap in people with MS and cognitive impairment is unknown. The primary objective of this study was to evaluate the relationship between fatigue and sleep quality, as measured by questionnaires, in a cohort of people with MS and mild to moderate cognitive impairment.

## 2. Methods

### 2.1. Subjects

The subjects in this study were 121 people with MS and cognitive impairment recruited for a 12-week randomized double-blind placebo controlled trial of Gingko Biloba for the treatment of cognitive impairment in MS [6]. This study analyzed baseline demographic and questionnaire data from this clinical trial. Two centers participated in the study, the Portland VA Medical Center and the VA Puget Sound Healthcare System (Seattle area). The IRB at each center approved the protocol and the study was registered with clinicaltrials.gov (NCT00841321) and all subjects provided written consent to participate. Subjects were required to have cognitive impairment (defined as ≥1 s.d. below the mean on the Stroop [7], Paced Auditory Serial Addition Test [7], Controlled Oral Word Association Test [8, 9], or California Verbal Learning Test [10, 11]) and not have severe depression (Beck Depression Inventory II (BDI II) score > 28) [9, 12]. Full details of inclusion and exclusion criteria have been published [6].

### 2.2. Measures

Scores on questionnaires assessing fatigue (Modified Fatigue Impact Score, MFIS [13]; Fatigue Severity Score, FSS [14]) and sleep quality (Pittsburg Sleep Quality Index, PSQI [15]) administered at the baseline visit were used for this analysis.

The MFIS is an MS specific 21-item questionnaire evaluating the impact of fatigue on cognitive, physical, and psychosocial functioning [13]. Scores on the MFIS range from 0 to 84, with higher scores indicating more fatigue. Previous studies have used cut-off scores of 38 or 45 on the MFIS to define fatigue [4, 5, 16–18]. It has been recommended that polysomnography be performed to diagnose sleep disorders in people with MS with an MFIS score over 34 [5]. The FSS is a 9-item questionnaire not designed specifically for people with MS which asks subjects to rate the degree to which fatigue impacts their lives [14]. Scores on the FSS range from 1 to 7, with lower scores indicating less fatigue. A cut-off score of greater than or equal to 4.6 on the FSS is suggested to discriminate between fatigued and nonfatigued subjects [14, 17, 19]. Both the MFIS and the FSS are validated and commonly used in treatment trials in MS [19–21].

The PSQI is a 19-item questionnaire about sleep quality [15]. The PSQI has seven subsections: subjective sleep quality, sleep latency, sleep duration, habitual sleep efficiency, sleep disturbances, use of sleep medications, and daytime dysfunction. Scores on the PSQI range from 0 to 21, with a score greater than or equal to five being associated with sleep disruption. The PSQI is well validated and has been widely used to study subjective sleep quality in healthy people and those with psychiatric and medical disorders, including MS [15, 22, 23].

### 2.3. Statistical Analysis

Multiple linear regression analysis was used to evaluate associations between fatigue (MFIS and FSS scores), sleep quality (PSQI global score), and demographics (age, disease duration, EDSS score, gender, and type of MS). This analysis was repeated using the PSQI subcomponent scores in place of the PSQI global score to determine if specific sleep issues were related to fatigue. Box-Cox transformation was performed on the FSS scores to ensure normal distribution. Post hoc analyses to evaluate the relationships between the depression (BDI II scores), the MFIS, FSS, and PSQI (global) scores were performed using Pearson correlations. All analyses were performed using SAS software (version 9.3).

## 3. Results

### 3.1. Demographics

The sample was typical of the population of people with MS, with a mean age of 52 years; 55% of the sample was female and 65% had relapsing remitting MS (Table 1). Eighty-six of the subjects reported taking an MS disease modifying therapy and of these 43 were taking a form of interferon*β*. I.

### 3.2. Sleep and Fatigue Measures

The mean MFIS score was 47.9 (sd = 15.1), the mean FSS score was 5.5 (sd = 1.2), and the mean global PSQI score was 13.0 (sd = 4.3). Fatigue as measured by the MFIS was significantly associated with decreased sleep quality (global PSQI), higher age, and greater disability (EDSS). Fatigue as measured by the FSS was significantly associated with decreased sleep quality (global PSQI) (Table 2).

Analysis using the PSQI component scores revealed that fatigue as measured by the MFIS was still associated with age and disability (EDSS) and was additionally correlated with PSQI component scores 1 (subjective sleep quality), 3 (sleep duration), and 7 (daytime dysfunction). The FSS scores were significantly correlated with PSQI component score 7 (daytime dysfunction), with correlations approaching significance for PSQI component scores 1 (subjective sleep quality, *P* = 0.06) and 3 (sleep duration, *P* = 0.05) (Table 3).

Subjects with severe depression (BDI II score > 28) were excluded from this study, and most subjects had no or minimal depression (90 of 121 subjects had BDI-II scores ≤13). However, given the known association between fatigue and depression [24], we also evaluated the correlation (Pearson's *r*) between the depression (BDI II score), fatigue, and sleep measures in this cohort. We found that the BDI II scores were significantly correlated with both fatigue measures (MFIS *r* = 0.48, *P* < 0.01; FSS *r* = 0.32, *P* = <0.01) and with the PSQI global score (*r* = 0.43, *P* < 0.01).

## 4. Discussion

This study is the first to demonstrate a relationship between self-reported fatigue and poor sleep quality in people with MS selected for cognitive impairment. This is consistent with previous studies reporting significant associations between self-reported fatigue and sleep quality, assessed by self-report or polysomnography, found in the general population of people with MS [5, 22, 23, 25–29].

Subjects in our study were selected for having MS and cognitive impairment but not specifically for being fatigued. Most subjects reported fatigue (FSS > 4.6) and poor sleep quality (PSQI > 5) and had minimal depression (BDI-II ≤ 13). The high incidence of fatigue in this sample is consistent with previous reports of MS-related fatigue [2, 3]. Fatigue (FSS and MFIS) and poor sleep quality (global PSQI) were strongly related. Specifically, the daytime dysfunction component of the PSQI was associated with FSS and MFIS scores and the sleep quality and sleep duration components of the PSQI were also significantly associated with MFIS scores. Several mechanisms may contribute to the relationship between fatigue and poor sleep in MS [27]. Increased levels of inflammatory cytokines or lesions in the brain may disrupt pathways involved with sleep and daytime alertness. Sleep apnea, which is common in people with MS, may also result in both fatigue and sleep disruption [28, 30, 31]. The use of MS disease modifying therapies, particularly interferon*β* preparations, may also contribute to both fatigue and sleep disturbance. The association between depressed mood and both sleep disturbance and fatigue found in this study suggests that depression may also be a mediator of poor sleep and MS-related fatigue [27, 32]. However, our exclusion of people with severe depression and the low incidence of depression in this sample limit our ability to evaluate these relationships.

Strengths of this study include its large sample size of roughly equal numbers of men and women that represent all four subtypes of MS. Furthermore, the subjects were not selected for fatigue or sleep problems, making our findings generalizable to a broad population of individuals with MS. Weaknesses of this study include that cognitive impairment could affect the subjects' ability to recall and accurately complete questionnaires about their experience of fatigue and sleep in the past weeks, the limited recruitment area, and the lack of objective measurement of sleep quality such as actigraphy or polysomnography. The study may have also benefitted from a control group of healthy volunteers or MS patients without cognitive deficits.

This study has a number of clinical implications. Typically, MS-related fatigue is treated with stimulant medications, energy efficiency techniques, and exercise, without assessment of sleep. This study shows that, for many individuals with MS, fatigue is associated with poor sleep quality. We therefore recommend that clinicians assess sleep in people with MS and fatigue by asking about subjective sleep quality, sleep duration, and daytime dysfunction and consider formal sleep evaluation with polysomnography. Treatment of diagnosed sleep problems should be pursued to reduce fatigue and improve quality of life in people with MS [22, 23, 33].

## Figures and Tables

**Table 1:** Demographics and sample characteristics for the 121 subjects.

Age (mean, SD, range)	52.1 years (9.1, 24–65)
Gender (%, *n*)	55% (66) females, 45% (55) males
Type of MS (%, *n*)	
Relapsing remitting	65% (78)
Secondary progressive	27% (33)
Primary progressive	7% (9)
Progressive relapsing	1% (1)
Disease duration (mean, SD, range)	20.1 years (11.7, 1–47)
EDSS* (mean, SD, range)	4.0 (1.92, 0–7.5)
BDI II* (mean, SD)	9.8 (6.7)
Cognitive testing* (mean, SD)	
Stroop	67.3 (7.5)
PASAT	25.9 (9.1)
COWAT	33.6 (10.3)
CVLT-II	−0.53 (1.19)

BDI II: beck depression inventory II, PASAT: Paced Auditory Serial Addition Test, COWAT: Controlled Oral Word Association Test, CVLT-II: California verbal learning test.

*Subjects were excluded from the study if they had severe disability (EDSS > 7.5) and significant depression (BDI II ≥ 28) or if they did not have significant cognitive impairment (greater than 1 s.d. less than mean on the Stroop, PASAT, COWAT, or CVLT-II).

**Table 2:** Associations between fatigue (MFIS and FSS scores), sleep quality (PSQI global score), and demographic characteristics.

	Fatigue scale			
	MFIS		FSS	
	F value	P	F value	P
Age (years)	6.98	**0.01**	0.08	0.78
Gender	0.74	0.39	0.22	0.64
Type of MS	0.01	0.93	1.93	0.17
EDSS	7.40	**0.01**	2.77	0.10
Disease duration	0.89	0.35	0.79	0.38
PSQI global	15.60	**<0.01**	12.09	**<0.01**

MFIS: Modified Fatigue Impact Scale, FSS: Fatigue Severity Scale, PSQI: Pittsburg Sleep Quality Index, EDSS: expanded disability status scale.

Bolded values indicate statistically significant associations.

**Table 3:** Associations between fatigue (MFIS and FSS scores), sleep quality components (PSQI component scores), and demographic characteristics.

	Fatigue scale			
	MFIS		FSS	
	*F* value	*P*	*F* value	*P*
Age years	7.71	**0.01**	0.12	0.73
Gender	0.08	0.78	1.25	0.27
Type of MS	0.02	0.89	2.69	0.10
Disease duration	1.36	0.25	0.80	0.37
EDSS	8.15	**0.01**	3.22	0.08
PSQI component scores				
(1) Sleep quality	5.76	**0.02**	3.68	0.06
(2) Sleep latency	0.04	0.83	0.00	0.98
(3) Sleep duration	6.36	**0.01**	3.77	0.05
(4) Sleep efficiency	1.47	0.23	0.47	0.50
(5) Sleep medications	0.66	0.42	0.22	0.64
(6) Sleep disturbances	0.12	0.73	0.44	0.51
(7) Daytime dysfunction	13.50	**<0.01**	4.60	**0.03**

MFIS: Modified Fatigue Impact Scale, FSS: Fatigue Severity Scale, PSQI: Pittsburg Sleep Quality Index, EDSS: expanded disability status scale.

Bolded values indicate statistically significant associations.
